# Investigating Post-translational Modifications in Neuropsychiatric Disease: The Next Frontier in Human Post-mortem Brain Research

**DOI:** 10.3389/fnmol.2021.689495

**Published:** 2021-07-16

**Authors:** Melanie J. Grubisha, Robert A. Sweet, Matthew L. MacDonald

**Affiliations:** ^1^Department of Psychiatry, University of Pittsburgh, Pittsburgh, PA, United States; ^2^Biomedical Mass Spectrometry Center, University of Pittsburgh, Pittsburgh, PA, United States

**Keywords:** proteomics, post-translational modification, schizophrenia, psychiatric disease, autism, post-mortem brain

## Abstract

Gene expression and translation have been extensively studied in human post-mortem brain tissue from subjects with psychiatric disease. Post-translational modifications (PTMs) have received less attention despite their implication by unbiased genetic studies and importance in regulating neuronal and circuit function. Here we review the rationale for studying PTMs in psychiatric disease, recent findings in human post-mortem tissue, the required controls for these types of studies, and highlight the emerging mass spectrometry approaches transforming this research direction.

## Introduction

Psychiatric disease imparts a substantial burden on the global population. For example, depression (Liu et al., 2020), schizophrenia (Charlson et al., 2018), bipolar disorder (Ferrari et al., 2016), and autism spectrum disorder (Baxter et al., 2015) are estimated to collectively impair the lives of over 350 million individuals across the globe, with limited treatment options and a relatively small number of compounds in FDA trials (O’Brien et al., 2014). Thus, psychiatric disease is deservedly the focus of intense scientific study. Psychiatric disorders have varying levels of heritability (Brainstorm et al., 2018) and genome wide association studies (GWAS) have identified risk loci for some, such as schizophrenia (Schizophrenia Working Group of the Psychiatric Genomics Consortium, 2014) and autism spectrum disorder (Grove et al., 2019). Studies in live patients [e.g., TMS, EEG (Vittala et al., 2020), and fMRI (Chen et al., 2011; Birur et al., 2017; Lau et al., 2019)] and of post-mortem brain tissue have found distinct impairments in discreet brain areas (Minzenberg et al., 2009), circuits (Eggan et al., 2012; Glausier et al., 2014; Lewis and Glausier, 2016), and cellular structures (Somerville et al., 2011; Shelton et al., 2015; Glausier et al., 2017; MacDonald et al., 2017; Uranova et al., 2018; McKinney et al., 2019) that could plausibly underlie disease symptoms. For example, individuals with schizophrenia display impairments in working memory tasks (Minzenberg et al., 2009), which are associated with altered activation of the dorsolateral prefrontal cortex (Minzenberg et al., 2009) as well as impairments in the processing of auditory sensory information (Javitt et al., 1995, 1997, 2000; Rabinowicz et al., 2000) which are associated with altered event-related potentials localized to the primary auditory cortex (Javitt et al., 1996; Lewis and Sweet, 2009). Alterations in dendritic spine density have been reproducibly observed in layer 3 of both the dorsolateral prefrontal cortex (Glantz and Lewis, 2000; Kolluri et al., 2005) and primary auditory cortex (Sweet et al., 2009; Shelton et al., 2015; MacDonald et al., 2017; McKinney et al., 2019) and are believed to contribute to the observed impairments in working memory and auditory sensory processing. It is important to note that the limited studies that have investigated layer 5 in a cortical region did not observe decreased spine density (Kolluri et al., 2005). Additionally, studies a have also not found a concurrent decrease in presynaptic boutons in layer 3 of cortical regions (Moyer et al., 2013). Thus, synaptic alterations in cortical regions in schizophrenia are limited to specific layers and structures. Interesting, subcortical regions appear to have distinct pathologies. For example, while the hippocampus displays similar decreases in dendritic spine density (Rosoklija et al., 2000), it displays different activity alterations and GABA cell pathology (Heckers and Konradi, 2015). Thus, it is essential to investigate the molecular pathology of individual brain areas, layers, cell types, and cellular structures. Alterations in dendritic spines have also been observed in bipolar disorder (Konopaske et al., 2014) and autism spectrum disorder (Martinez-Cerdeno, 2017).

While many areas of research benefit greatly from the use of animal models, polygenetic psychiatric disorders are difficult, if not impossible to model in animals, complicating the investigation of disease etiology. In an effort to elucidate the molecular mechanisms driving these structural and functional impairments the field has turned to transcriptomic and proteomic analyses of human post-mortem brain tissue to quantify disease associated differences in transcripts (Hernandez et al., 2021) and proteins (Martins-de-Souza, 2012; Focking et al., 2015; MacDonald et al., 2019b), providing many valuable insights into disease pathology.

More recently, multi-omics analyses, grounded in GWAS, have identified quantitative trait loci (QTLs) for common risk variants associated with gene expression (eQTLs) (Gandal et al., 2018) and protein levels (pQTLs) (Robins et al., 2019). eQTL studies have started to provide insight into the biological effects of common risk loci. To date at least one human brain pQTL study has been published, finding that only a subset of pQTLs were also eQTLs (Robins et al., 2019), highlighting the disconnect between the transcriptome and the proteome. Several groups are currently pursuing well powered proteomic investigations of the human brain, suggesting that additional proteogenomic studies will further map associations between common variants and protein expression in psychiatric disease. While these studies are powerful, they fail to capture the more dynamic aspects of the proteome, such as post-translational modifications (PTMs), that are among the final mediators of cell and circuit activity, and are difficult, if not impossible, to infer from the transcript or protein levels.

Post-translational modifications have been studied in neurological disorders, most notably Tau hyperphosphorylation in Tauopathies such as Alzheimer’s disease (Avila, 2006; Neddens et al., 2018), but they have received less attention in psychiatric disease and their study in human post-mortem brain tissue is often viewed with skepticism. This skepticism is not entirely unwarranted, as many PTMs are highly dynamic. However, many are stable post-mortem and, as PTMs regulate protein activity, this information is likely more valuable than protein levels alone. Here we review the rationale for studying PTMs in psychiatric disease, recent findings in human post-mortem brain tissue, the common pitfalls and required controls for these types of studies, and highlight the emerging mass spectrometry approaches transforming this research direction.

## PTMs Are Implicated in Psychiatric Disease

Unbiased genetic analyses firmly implicate PTMs in the etiology of a number of psychiatric diseases. The most recent report from The Schizophrenia Working Group of the Psychiatric Genomics Consortium et al. (2020) identified 130 genes with common non-coding variation associated with schizophrenia, including seven protein kinases/phosphatases (AKT3, MOB4, DCLK3, PTPRK, PAK6, FHIT, and MAPK3), a proteasome subunit (PSMA4), and a ubiquitin ligase (PJA1) (The Schizophrenia Working Group of the Psychiatric Genomics Consortium et al., 2020). Similarly, of the 209 genes currently implicated in Autism Spectrum Disorder with high confidence (as currently defined by SFARI), 16 are kinases/phosphatases (BCKDK, BRSK2, CASK, CDKL5, CSNK2A1, DMPK, DYRK1A, PPP2R5D, PPP1R9B, PPP5C, PTEN, PTK7, PTPN11, TAOK1, TLK2, and TEK), three are ubiquitin ligases (HECTD4, UBE3A, and UBR1), and one is a proteasome subunit (PSMD12) (Foundation SFARI, 2021).

Investigations of transcript levels in human post-mortem brain tissue from autism spectrum disorder and schizophrenia subjects further implicate PTMs. The most recent meta-analysis of RNAseq studies from PsychENCODE (Gandal et al., 2018) found that 52 protein kinases, 14 protein phosphatases, 9 proteasome subunits, and 22 ubiquitin ligases were differentially expressed in autism spectrum disorder (Gandal et al., 2018); while 123 protein kinases, 41 protein phosphatases, 7 proteasome subunits, and 62 ubiquitin ligases were differently expressed in schizophrenia (Gandal et al., 2018).

These findings are not surprising as schizophrenia and autism spectrum disorder are both widely viewed as developmental synaptopathies (Grant, 2012; Won et al., 2013; Washbourne, 2015; Guang et al., 2018) and a multitude of studies have demonstrated the essential roles of protein phosphorylation and the ubiquitin-proteasome system in synaptic plasticity, long term potentiation, and learning (Roche et al., 1994; Lee, 2006; Mabb and Ehlers, 2010; Kwon and Sabatini, 2011; Woolfrey and Dell’Acqua, 2015; Hegde, 2017). As stated above, while these genetic and transcriptomic investigations can, and have, implicated specific classes of PTMs and enzymes, they cannot capture their effects on the broader synaptic proteome. Furthermore, while altered levels of a given kinase or ubiquitin ligase can be modeled in cell culture or animal models, the complex genetic risk factors and environmental circumstances that give rise to psychiatric disease, as well as the unique circuitry and neuronal populations of the human brain, cannot. It is important to note here that neurons and organoids derived from patient pluripotent stem cells can mimic the genetic risk profiles of psychiatric disease and provide a powerful window into pathological neurodevelopmental processes (Brennand et al., 2011; Khakipoor et al., 2020; Marton and Pasca, 2020). However, these models still lack the longevity of human adolescence and adulthood as well as interactions with systemic features (e.g., circulating hormones or the microbiome) and environmental risk factors. Thus, post-mortem brain studies are essential to investigating the molecular changes associated with psychiatric disease. In next section we will seek to answer the following questions: Can disease associated PTM differences be observed in human post-mortem brain tissue and do these PTMs have biological validity. The history of Tau gives us some hope that PTMs observed in human post-mortem brain tissue can yield insights into disease etiology (Simic et al., 2016).

## A Brief History of Modern PTM Studies in Schizophrenia and Other Psychosis Related Disorders

By the early 2000s dendritic spines (Glantz and Lewis, 2000) and NMDA receptors (Tsai et al., 1998; Thaker and Carpenter, 2001) had been implicated in schizophrenia pathology. As the decade progressed, genetic, transcriptomic, and early mass spectrometry studies continued to implicate postsynaptic ligands, receptors, and scaffolding proteins, such as ERBB4 (Silberberg et al., 2006), AKT (Matsubara et al., 2001; Emamian et al., 2004a; Ikeda et al., 2004; Turunen et al., 2007), NRG1 (Stefansson et al., 2002, 2003; Williams et al., 2003; Yang et al., 2003), and PSD95 (Ohnuma et al., 2000) in schizophrenia. Several groups then began utilizing traditional antibody-based approaches and eventually mass spectrometry for targeted phospho-analyses in patient tissue.

Emamian et al. (2004b) found that phosphorylation of NR1 S897 was decreased (while total NR1 levels were unaltered) in frontal cortex tissue from individuals with schizophrenia. This site was of particular interest as antipsychotic drugs were known to increase NR1 S897 phosphorylation in primary neuronal cultures (Leveque et al., 2000) and S897 is essential for antipsychotic drug-mediated gene expression (Leveque et al., 2000). A later study further demonstrated S897’s importance in NMDAR synaptic incorporation, NMDAR-mediated synaptic transmission, AMPA receptor mediated synaptic transmission, and long-term potentiation (Li et al., 2009). Pinacho et al. (2015) found that S770 phosphorylation on the transcription factor SP4 was positively correlated with negative symptoms in schizophrenia subjects. Importantly, they also found that SP4 phosphorylation was inversely correlated with SP4 levels. SP4 regulates dendritic arborization (Ramos et al., 2007) and phosphorylation at SP4 S770 regulates stability of the protein (Pinacho et al., 2015). More recently, Vanderplow et al. (2021) found that phosphorylation of PI3K, AKT, and MTOR was decreased in cortical tissue from a subset of subjects with bipolar disorder. Subsequent studies in mice found that overexpression of dominant negative AKT impaired dendritic spine maintenance and performance in cognitive tasks (Vanderplow et al., 2021).

Finally, Grubisha et al. (2021) used mass spectrometry-based proteomics to investigate 18 phosphorylation sites on MAP2 in cortical tissue from schizophrenia subjects, finding differential phosphorylation at 9 while total levels of MAP2 were unaltered. Generating a transgenic mouse containing a phosphomimetic mutation at S1782 (S1782E) they found reductions in basilar dendritic length and complexity along with reduced spine density (Grubisha et al., 2021).

The studies above measured static phosphorylations, presumably preserved at death. But a few adventurous groups have pushed these studies further, attempting to capture dynamic phosphorylation activities in human post-mortem brain tissue. In two publications, Hahn and Wang combined targeted phosphorylation studies with a post-mortem tissue-stimulation paradigm to identify phosphorylation differences in schizophrenia after receptor stimulation (Hahn et al., 2006; Wang et al., 2020). In the first study they found that NRG1 stimulation of ERBB4 decreased glutamate/glycine induced phosphorylation of NMDAR2A and PLCγ, likely driven by increased association between ERBB4 and PSD95, but independent of the levels of any of the assayed proteins (Hahn et al., 2006). In the second study, they observed increased phosphorylation of mGluR5 after stimulation, which was accompanied by decreased coupling with Gq/11, indicating decreased mGluR5 activity, again independent of the levels of any of the assayed proteins. Taking a different approach, McGuire et al. (2014, 2017) utilized kinase arrays to interrogate signaling cascades in cortical tissue from schizophrenia subjects, finding significant alterations in kinome activity that further implicate cellular and ion homeostasis as well as cytoskeletal organization in schizophrenia.

A key driver underlying the incomplete correlation between mRNA and protein abundance is the fact that protein turnover is dynamic. The principal mechanism of turnover is the ubiquitin-proteasome system in which polyubiquitinated proteins are targeted to the proteasome for degradation. Additionally, the ubiquitin-proteasome system regulates synaptic protein stability and is essential for LTP and learning (Mabb and Ehlers, 2010; Hegde, 2017). Thus, while it has received less attention than phosphorylation, the ubiquitin-proteasome system is beginning to be investigated in schizophrenia. Rubio et al. (2013) first observed differences in both free and protein ubiquitination. More recently, Nucifora et al. (2019) found that increased protein ubiquitination was correlated with increased protein insolubility in cortical tissue from schizophrenia subjects. Finally, paralleling the kinome arrays used to assess kinase activity in schizophrenia, Scott and Meador-Woodruff (2020) utilized proteasome activity assays, finding altered trypsin and chymotrypsin like activity in schizophrenia tissue.

While this review has focused on ubiquitination and phosphorylation a growing body of work implicates additional PTMs such as glycosylation and myristylation in schizophrenia, reviewed in detail in Mueller and Meador-Woodruff (2020). Briefly, alterations in N-Glycosylation on GABA (Mueller et al., 2014), NMDA (Tucholski et al., 2013b), and AMPA (Tucholski et al., 2013a) receptors have been observed in schizophrenia.

The studies reviewed above suggest several points: (1) That differences in PTMs can be observed in brain tissue from subjects with psychiatric disease; (2) that levels of multiple classes of PTMs are altered across multiple protein families; and (3) when tested in forward genetic models, individual PTMs can significantly impact disease relevant biology such as glutamatergic signaling and dendritic spine plasticity, that could not be predicted by genetics, transcriptomics, or even protein quantification. For example, MAP2 is not found at any schizophrenia risk loci and its protein levels are unaltered in schizophrenia tissue, yet it is hyperphosphorylated at multiple sites in schizophrenia and modeling just one of these sites induces a loss of dendritic spines.

The breadth of the PTM alterations observed in schizophrenia *via* mostly targeted approaches highlights the need for broad and systematic investigations of PTMs in psychiatric disease. Specifically, next generation studies should be rigorously designed to catalog post-mortem effects on individual PTM sites, be performed in well powered and well-balanced cohorts, utilize state-of-the-art mass spectrometry approaches, target selected brain areas, cortical layers, cell types, and microdomains, and take advantage of new informatic and statistical approaches to multi-omic integration to map associations between genes, multiple PTMs, and phenotypes.

## Experimental Considerations for Investigating PTMs in Post-Mortem Brain Tissue

The impact of post-mortem interval (PMI; the time between when a subject becomes deceased and the brain tissue is fixed and/or frozen) on molecular integrity has long been appreciated and three main strategies have been employed to account and control for this confound. (1) The effect of PMI on individual PTM sites can be modeled, using either mouse (MacDonald et al., 2019b) or human (Gallego Romero et al., 2014; Jaffe et al., 2017) tissue. Several groups have used this approach to either correct for mRNA degradation (Jaffe et al., 2017) in human studies or to identify proteins that degrade non-linearly (MacDonald et al., 2019b) across PMI for removal from case-control statistical comparisons. The same approach should be employed in PTM studies in human post-mortem brain tissue, identifying which specific modifications at which sites degrade non-linearly over time. (2) PMI is often included as a co-factor in statistical analysis. (3) When possible, diagnostic groups or subject pairs should be matched as closely as possible for PMI [as well as other factors that are known to impact proteins and PTMs such as sex (Bangasser et al., 2017) and age (Carlyle et al., 2017)]. Given the dynamic nature of many PTMs, all of these approaches should be utilized, and it is especially important to identify PTMs that are rapidly degraded early in PMI and to remove them from downstream statistical analyses. In the past, generating a well-balanced and powered cohort was a significant challenge, but with the recent unification of multiple brain tissue repositories under the aegis of the NIH NeuroBioBank, researchers now have access to quality tissue from thousands of well cataloged cases and appropriate controls.

## Emerging Mass Spectrometry Approaches for PTM Quantification in Post-Mortem Brain Tissue

Advances in mass spectrometry instrumentation and sample preparation techniques continue to increase the throughput, breadth and depth of PTM coverage, and some of these approaches have begun to see use in human post-mortem brain tissue. Next generation proteomics instruments such as the timsTOF (Bruker) and Orbitrap instruments (ThermoFisher) with increased scan speeds facilitate deep coverage of modified peptides. For example, Ping et al. (2020) utilized a tandem mass tag (TMT) based approach to quantify over 48,000 phosphopeptides (representing over 33,000 unique phosphorylation sites) in human post-mortem brain tissue. In addition to increased instrument speed and sensitivity, modern mass spectrometers now offer an array of dissociation methods (e.g., CID, HCD, and ETD) enhancing the identification, and subsequent quantification, of high energy and complex PTMs, such as phosphorylation (Jedrychowski et al., 2011; Potel et al., 2019) and glycosylation (Reiding et al., 2018; Riley et al., 2020).

Protocols to enrich phosphopeptides and glycopeptides from tryptic digests are now well developed and can be accomplished with high efficiency using commercially available kits and specialized liquid handling robots with pre-programmed proteomic applications, such as the AssayMAP BRAVO (Agilent), enabling both throughput and quantitative depth. Quantification of protein ubiquitination initially proved more challenging in standard proteomic work flows as trypsin digests off the larger ubiquitin side chain leaving only a lysine bound gly-gly which was difficult to capture. However, the recent availability of commercial antibodies and refined sample preparation now allow for deep profiling of protein ubiquitination in human samples as well; for example, over 14,000 ubiquitination sites were recently quantified in human tumor cells (Udeshi et al., 2020).

## The Spatial Resolution Limits of PTM Quantification in the Human Brain

As reviewed above, the activity, structure, and molecular pathologies associated with psychiatric illness are highly spatially localized, displaying brain area, layer, cellular, and microdomain specificity. Multiple groups have used mass spectrometry to quantify protein levels in cortical layer captures (Pabba et al., 2017; MacDonald et al., 2019a) and synaptic microdomains obtained by either biochemical fractionation (Chang-Gyu et al., 2009; MacDonald et al., 2012, 2019b; Focking et al., 2015) or Fluorescence-activated Cell Sorting (Gylys et al., 2004; Sokolow et al., 2012) from human post-mortem brain tissue. Synaptic microdomain enrichments generated by biochemical fractionation can provide sufficient material for phosphopeptide enrichment and quantification (Trinidad et al., 2008) which has been accomplished in fresh human brain tissue (DeGiorgis et al., 2005). While it has not yet been demonstrated in human brain tissue, laser capture microdissection and Fluorescence-activated Cell Sorting both likely generate sufficient material for investigation of the phosphoproteome with the aid of isobaric labeling such as TMT and iTRAQ. Glycopeptide enrichment is sufficiently robust that it could likely be applied in the same scenarios as phosphopeptides. Conversely, ubiquitin-motif peptide enrichment still requires significant amounts of starting material, likely limiting its application to brain areas for the present. Finally, while recent advances in single cell proteomics (Levy and Slavov, 2018) such as SCoPE-MS (Budnik et al., 2018) now allow for the quantification of hundreds of proteins in single cells, deep mass spectrometry based quantification of PTMs is still in the future. Given the pace of instrument development, single cell PTM quantification is likely not too far off.

## Informatics

Keeping a pace with the advancements in instrumentation, the last few years has seen the release of multiple software packages for exploring PTMs in the context of known kinase-substrate relationships, kinase motifs, protein–protein interactions, and protein networks. Packages such as iGPS (Song et al., 2012) and KEA2 (Lachmann and Ma’ayan, 2009) identify known or presumptive kinase-substrate interaction from phosphoproteomics data, potentially identifying upstream kinases driving observed changes in protein phosphorylation. Other tools such as ProteoViz (Storey et al., 2020) integrate the identification of sequence motifs and kinases with gene set enrichment pathway analysis while CausalPath “identifies potentially causal dependencies between measured protein features (such as phosphorylations or global protein levels) using literature-curated biological pathways” (Babur et al., 2021). Thus, researchers in the PTM space have a rapidly expanding number of informatics resources to draw upon in exploring their datasets.

## Closing Thoughts

In closing, PTMs are implicated in the etiology of neuropsychiatric diseases by unbiased genetic and transcriptomic studies, most notably autism spectrum disorder and schizophrenia. When investigated in human post-mortem brain tissue using targeted approaches, robust alterations in phosphorylation, glycosylation, and ubiquitination are observed in psychiatric disease, suggesting a much broader set of changes, with likely associations between different PTMs as well as the genome, transcriptome, and proteome. Advances in mass spectrometry instrumentation and proteomic sample preparation methods now allow for sufficiently powered studies to map these interactions, which, when combined with emerging informatics tools, will surely provide insight into the etiology of many psychiatric diseases as PTMs are the ultimate mediators of so many neuronal and circuit activities.

## Author Contributions

MM wrote the first draft of the mini review. MG, RS, and MM revised and reviewed the second draft of the mini review. All authors contributed to the article and approved the submitted version.

## Conflict of Interest

The authors declare that the research was conducted in the absence of any commercial or financial relationships that could be construed as a potential conflict of interest.
